# Supplementary material for: Selective Extracellular Signal-Regulated Kinase 1/2 (ERK1/2) Inhibition by the SCH772984 Compound Attenuates In Vitro and In Vivo Inflammatory Responses and Prolongs Survival in Murine Sepsis Models
Source: Int J Mol Sci. 2021 Sep 22;22(19):10204. doi: 10.3390/ijms221910204 (PMC8508766; doi:10.3390/ijms221910204)

Figure S4: Effects of SCH772984 (ERKi) compound on ERK1/2 phosphorylation 6h and 12h post CLP procedure. Tissue samples were disrupted in RIPA buffer, lysates were resolved by SDS-PAGE and electrotransferred to PVDF membrane and immunostained with indicated antibodies. The graph (lower panel) shows averaged pERK1/2 abundance normalized to Actb by densitometry analysis of western blotting bands. Means (+/- SD) are presented.

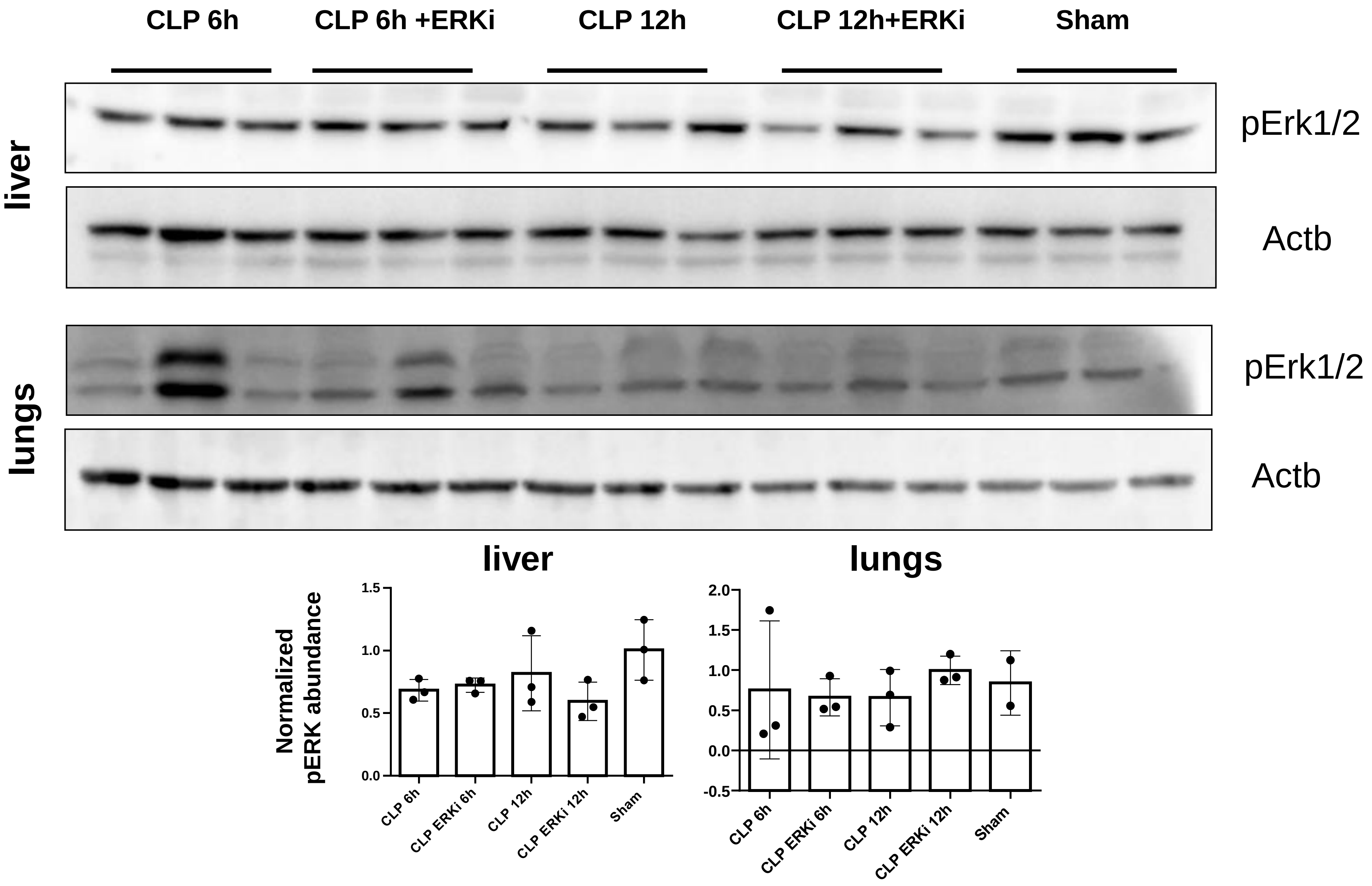

Supplement: Supplementary file 1 [file ijms-22-10204-s001.zip › Figure S4.pdf]
