# Supplementary material for: Selective Extracellular Signal-Regulated Kinase 1/2 (ERK1/2) Inhibition by the SCH772984 Compound Attenuates In Vitro and In Vivo Inflammatory Responses and Prolongs Survival in Murine Sepsis Models
Source: Int J Mol Sci. 2021 Sep 22;22(19):10204. doi: 10.3390/ijms221910204 (PMC8508766; doi:10.3390/ijms221910204)

Figure S3. Plasma levels of indicated cytokines were measured with 26-Plex Mouse ProcartaPlex immunoassay in CLP, SCH772984 (ERKi) treated CLP, and sham-operated animals (n ≥ 6) at 6 and 12 hours endpoint. Showed are 19 analytes that were not significantly changed in CLP vs CLP+ERKi comparison.

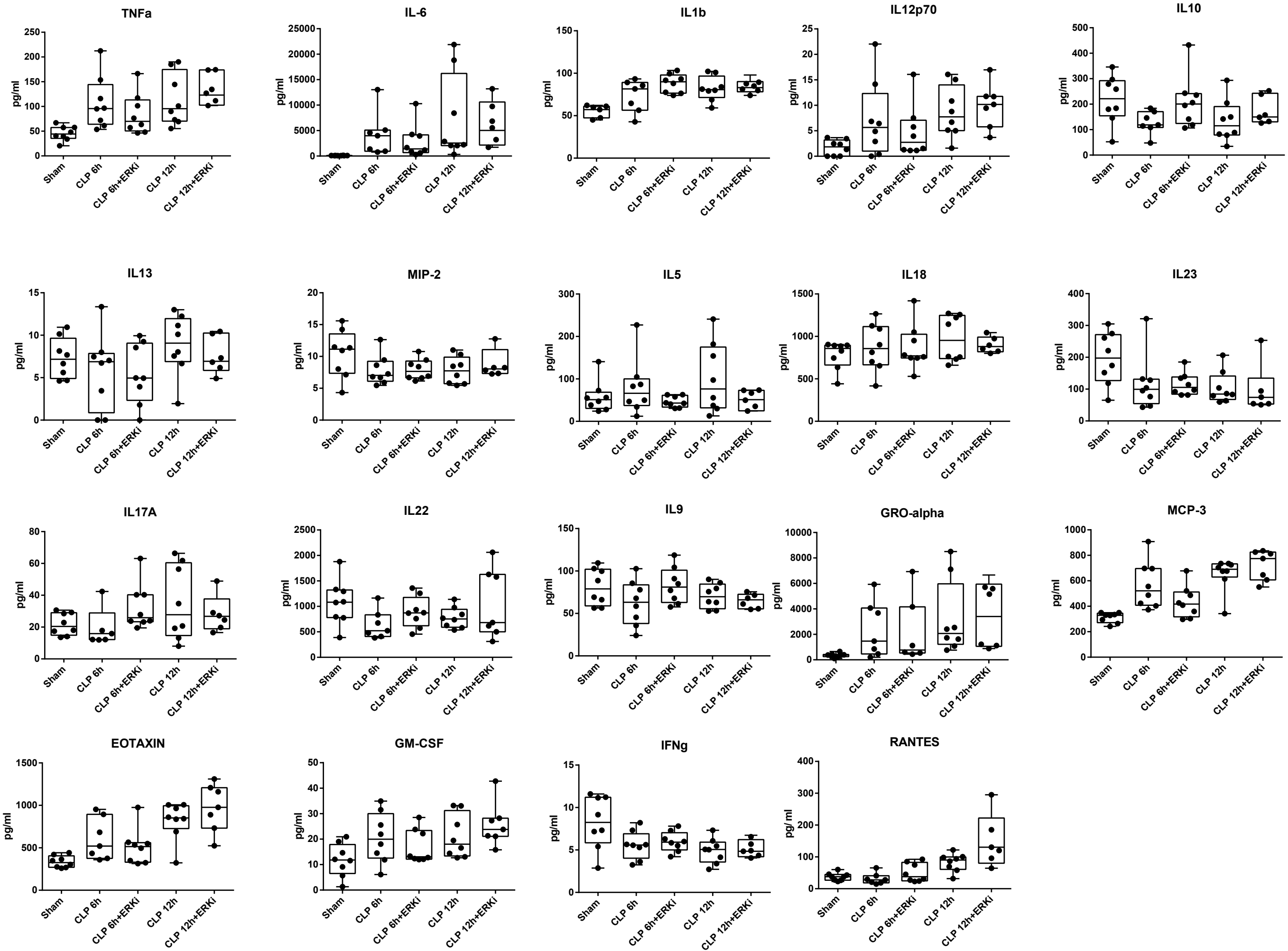

Supplement: Supplementary file 1 [file ijms-22-10204-s001.zip › Figure S3.pdf]
