# Supplementary material for: Selective Extracellular Signal-Regulated Kinase 1/2 (ERK1/2) Inhibition by the SCH772984 Compound Attenuates In Vitro and In Vivo Inflammatory Responses and Prolongs Survival in Murine Sepsis Models
Source: Int J Mol Sci. 2021 Sep 22;22(19):10204. doi: 10.3390/ijms221910204 (PMC8508766; doi:10.3390/ijms221910204)

Figure S1: The results of TNFa release in four murine cell lines following lipopolysaccharide administration using AlphaLISA assay. Cells were maintained under the recommended conditions and were stimulated with 10 µg/ml LPS for 4 and 24 hours. At indicated timepoints levels of TNFa were measured in medium AlphaLISA assay (Perkin Elmer). Data represent mean ± SD of 12-plicates.

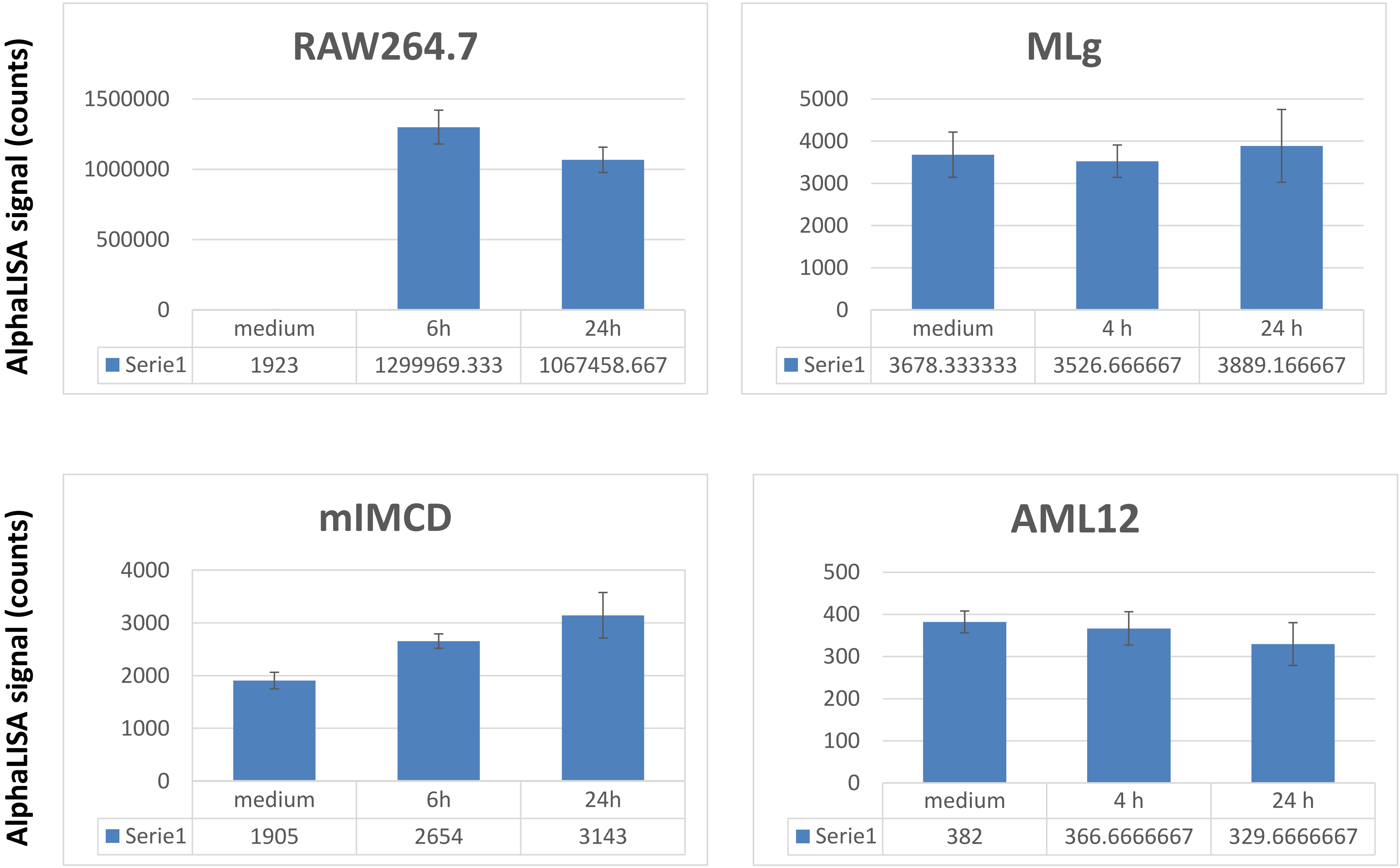

Supplement: Supplementary file 1 [file ijms-22-10204-s001.zip › Figure S1.pdf]
