# Supplementary material for: Selective Extracellular Signal-Regulated Kinase 1/2 (ERK1/2) Inhibition by the SCH772984 Compound Attenuates In Vitro and In Vivo Inflammatory Responses and Prolongs Survival in Murine Sepsis Models
Source: Int J Mol Sci. 2021 Sep 22;22(19):10204. doi: 10.3390/ijms221910204 (PMC8508766; doi:10.3390/ijms221910204)

Figure S2: Chromatin accessibility at TNF locus following the time course of LPS challenge (blue) and in presence of SCH772984 (ERKi, red). The ATAC-Seq tracks from two biological replicates are shown.

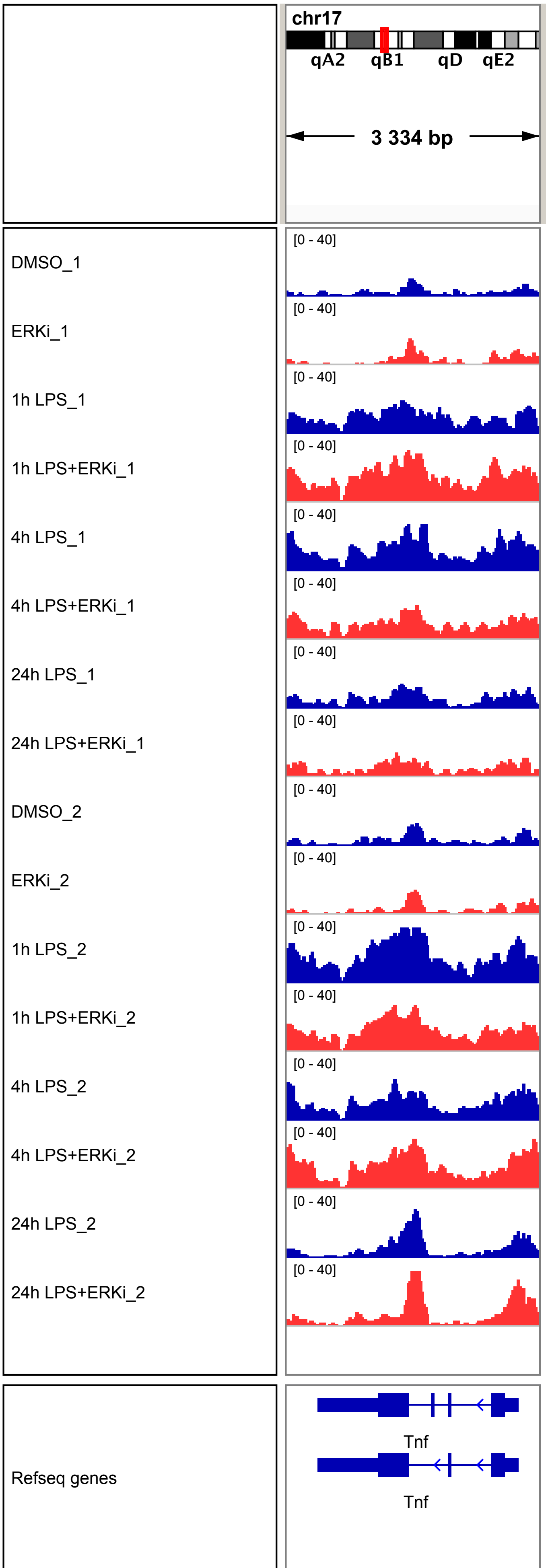

Supplement: Supplementary file 1 [file ijms-22-10204-s001.zip › Figure S2.pdf]
